# Structural characteristics, antioxidant and hypoglycemic activities of polysaccharides from *Mori Fructus* based on different extraction methods

**DOI:** 10.3389/fnut.2023.1125831

**Published:** 2023-04-06

**Authors:** Yuanyuan Huang, Wen Xie, Ting Tang, Huaguo Chen, Xin Zhou

**Affiliations:** ^1^Key Laboratory for Information System of Mountainous Areas and Protection of Ecological Environment, Guizhou Normal University, Guiyang, China; ^2^Guizhou Engineering Laboratory for Quality Control & Evaluation Technology of Medicine, Guizhou Normal University, Guiyang, China

**Keywords:** *Mori Fructus* polysaccharide, extraction methods, structural, antioxidant, hypoglycemic

## Abstract

The mulberry (*Mori Fructus*), which is rich in many nutrients needed by the human body, serves as both food and medicine. Polysaccharides, which are considered to be important pharmacological components of mulberry, have received a lot of study for their structure and biological activity. In this study, six mulberry fruit polysaccharides (MFPs) were extracted by different extraction methods, and their physicochemical structures, antioxidant, and hypoglycemic biological activities were investigated and compared. According to the findings, MFP-III exhibited the best α-glucosidase and α-amylase inhibition, whereas MFP-IV had the strongest scavenging activity against DPPH and ABTS. Scanner electron microscopy (SEM) and high-performance liquid chromatography (HPLC) analysis showed that the apparent morphology and monosaccharide content of MFP were significantly impacted by the different extraction techniques. The results of experiments using Congo red, Fourier transform infrared spectroscopy (FT-IR), nuclear magnetic resonance (NMR), thermogravimetric analysis (TG), and the Congo red experiment showed that the MFP functional groups, glycosidic bonds, triple helix structure, and thermal stability were not significantly different between the extraction methods. According to the aforementioned research, various extraction methods had different effects on the chemical composition and biological activity of mulberry polysaccharides. This information can provide a scientific basis for selecting suitable extraction methods to obtain mulberry polysaccharides with ideal biological activity.

## 1. Introduction

Polysaccharide is a class of complex and bulky carbohydrates composed of more than ten monosaccharide molecules connected by glycosidic bonds, which is widely found in animal cell membranes and cell walls of plants (1). Some polysaccharides from animals, plants, and microorganisms have been demonstrated to have significant biological activities in recent years due to the intensification of research on polysaccharides, including anti-inflammatory, antioxidant, hypoglycemic, hypolipidemic, antitumor, and antiviral effects (2). Due to their numerous origins, low toxicity, and nutritional health benefits, polysaccharides have increasingly become a hot topic in functional food research.

Mulberry (*Mori Fructus*) is the mature fruit of *Morus alba* L., a plant of the Moraceae family, widespread in Asia, Europe, South America, and North America (3). It has important medicinal and nutritional value (4). Mulberry is a beneficial fruit for maintaining one’s health and beauty maintenance since modern pharmacological investigations have revealed that it primarily has the benefits of reducing liver damage, enhancing vision, being anti-radiation, anti-oxidative, anti-aging, and boosting blood cell growth (5). Mulberry includes a wide range of nutrients and active substances, including flavonoids, polysaccharides, and vitamins. Polysaccharides in particular are rich in content and have considerable pharmacological actions as well as beneficial antioxidant, hypoglycemic, and hepatoprotective properties (6).

It was discovered that the structure of mulberry polysaccharide produced using various extraction methods varied, and the degree to which the variation in structure would impact its biological activity varied. However, their extraction processes typically result in low yields, ruined biological processes, and expensive energy usage. Furthermore, there aren’t many comprehensive data on how various extraction methods affect the physicochemical characteristics and bioactivity of mulberry polysaccharides.

**FIGURE 1:**
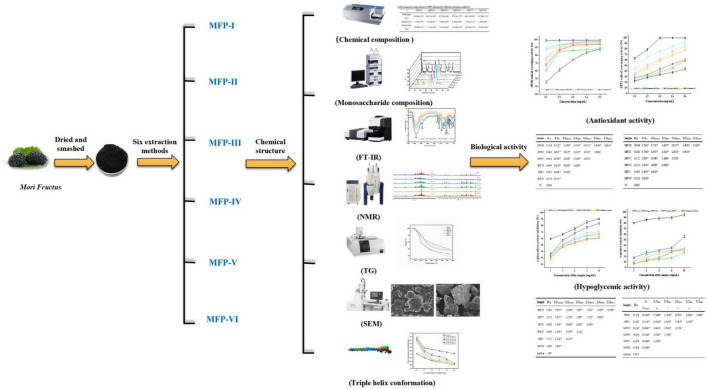
Structural characterization and bioactivity of different fractions of mulberry polysaccharides.

Mulberry polysaccharide is a polar macromolecule compound, which is easily soluble in water, and aqueous solvent-assisted extraction is a green and simple method for the dissolution of plant polysaccharides. Therefore, in this study, six MFPs were extracted using cold water extraction, boiling water extraction, ultrasound-assisted extraction with various polar waters (50, 75, and 100%), and hot water (80°C) extraction methods in order to more thoroughly investigate the effects of different extraction methods on the physicochemical properties and biological activities of MFP. The polysaccharides extracted by the six methods were contrasted in terms of their chemical composition, monosaccharide composition, IR spectra, NMR, thermal stability, microstructure, triple helix structure, antioxidant activity, and hypoglycemic activity (shown in Figure 1). The findings of this study will serve as a scientific foundation for the choice of appropriate extraction method to obtain MFP with desired biological activities, as well as for the precise development and utilization of MFP. They may also serve as a basis for the development of new drugs and health food products that use MFP as raw materials.

## 2. Materials and methods

### 2.1. Raw materials

The dried mulberry (product lot No.: 200802) was purchased from Sichuan Borin Pharmaceutical Co., Ltd. The fruits were crushed and sieved through 40 mesh to obtain powdered samples.

### 2.2. Biochemical reagents and standards

2,2-Diphenyl-1-picrylhydrazyl (DPPH), 2,2-azino-bis (3-ethyl-benzothiazole-6-sulfonic acid) (ABTS), α-amylase, α-glucosidase, p-nitro-α-D-glucopyranoside (PNPG), acarbose, and were purchased from Beijing Solarbio (Beijing Solarbio Science & Technology Co., Ltd.). Monosaccharide standards: galactose, galacturonic acid, glucuronic acid, etc., (purity ≥98%) were purchased from Guizhou Dida Technology Co. All other chemicals and solvents used were of analytical grade. Distilled water was used for polysaccharide extraction and characterization process, while ultrapure water was used for bioassay.

### 2.3. Extraction of polysaccharide from mulberry fruit

#### 2.3.1. Pre-treatment of mulberry fruit

**FIGURE 2:**
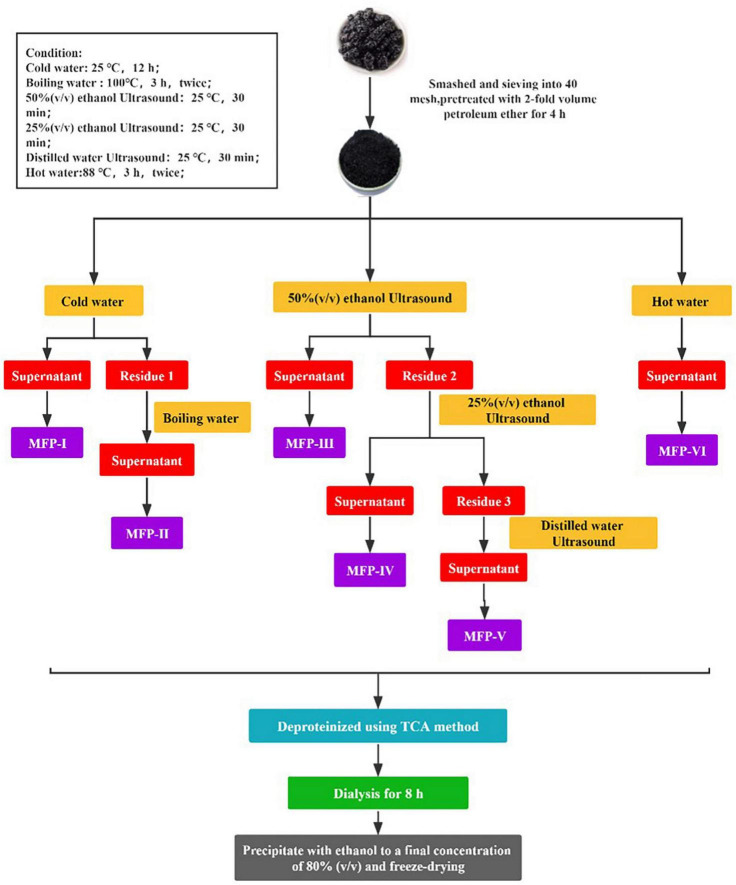
Different extraction methods of mulberry polysaccharides.

A total of 2 kg of dry mulberry fruit powder was mixed with 4000 mL of petroleum ether at a material-to-liquid ratio of 1:2 (g/mL) to remove fat-soluble impurities. The residue was separated by filtration and dried to obtain the pre-treated dry sample. Extracted according to the method shown in Figure 2.

#### 2.3.2. Cold water extraction (MFP-I)

After defatting, mulberry samples were added to distilled water at a ratio of 1:15 (g/mL) and left to stand at room temperature for 12 h. At the end of extraction, the supernatant was collected by centrifugation (3500 r/min, 10 min). The residue was dried and set aside, named residue 1. Finally, the supernatant was concentrated to 1/4 of the original volume to obtain MFP-I.

#### 2.3.3. Boiling water extraction (MFP-II)

The dried residue 1 was added to distilled water at a ratio of 1:15 (g/mL) and extracted twice with boiling water at 96–98°C for 3 h. At the end of extraction, the supernatant was collected by centrifugation and the residue was discarded. Finally, the supernatant was concentrated to 1/4 of the original volume to obtain the MFP-II.

#### 2.3.4. 50%(v/v) ethanol ultrasound extraction (MFP-III)

After defatting, mulberry was added to 50% (v/v) anhydrous ethanol at a ratio of 1:15 (g/mL) and extracted twice with ultrasound for 30 min each time. At the end of extraction, the supernatant was collected by centrifugation. The residue was dried and named residue 2. Finally, the supernatant was concentrated to 1/4 of the original volume to obtain MFP-III.

#### 2.3.5. 25%(v/v) ethanol ultrasound extraction (MFP-IV)

The dried residue 2 was added to 25% (v/v) anhydrous ethanol at a ratio of 1:15 (g/mL) and extracted twice with ultrasound for 30 min each time. At the end of extraction, the supernatant was collected by centrifugation. The residue was dried and named residue 3. Finally, the supernatant was concentrated to 1/4 of the original volume to obtain MFP-IV.

#### 2.3.6. Water ultrasound extraction (MFP-V)

The dried residue 3 was added to distilled water in the ratio of 1:15 (mg/mL) and extracted twice with ultrasound for 30 min each time. At the end of extraction, the supernatant was collected by centrifugation and the residue was discarded. Finally, the supernatant was concentrated to 1/4 of the original volume to obtain MFP-V.

#### 2.3.7. Hot water extraction (MFP-VI)

The defatted mulberry was added to distilled water at a ratio of 1:15 (mg/mL) and extracted twice in hot water at a constant temperature of 80°C for 3 h. After the extraction, the supernatant was collected by centrifugation and the filter residue was discarded. Finally, the supernatant was concentrated to 1/4 of the original volume to obtain MFP-VI.

#### 2.3.8. Removal of protein

Deproteinization of mulberry polysaccharides by trichloroacetic acid (TCA) method. The above six components of mulberry polysaccharide extract were added with 3% (v/v) TCA at a mass ratio of 1:1 (g/g), shaken well for 20 min, and left to stand at 4°C under refrigeration for 4 h. Then the supernatant was collected by centrifugation (4000 r/min, 10 min), concentrated, and the deproteinization was repeated for 2 times to save the final concentrated solution.

#### 2.3.9. Removal of small molecules

The above six component deproteinized mulberry polysaccharide solution was dialyzed in a dialysis bag with a cut-off molecular weight of 8000–14000 Da for 8 h. Then, the supernatant was collected by centrifugation (4000 r/min, 10 min) and concentrated.

#### 2.3.10. Ethanol precipitation

Four times the volume of anhydrous ethanol was added to 2.3.9 purified mulberry polysaccharide extracts to a final ethanol concentration of 80% and left at 4°C for 24 h. Then centrifuged (3500 r/min, 10 min), the precipitate was re-solubilized, ethanol precipitated again, and repeated twice. The precipitates were combined, washed 3 times with anhydrous ethanol and acetone in turn, and freeze-dried under a vacuum to obtain purified mulberry polysaccharide.

Each sample after freeze-drying was weighed and its yield was calculated according to the following formula:


$$\mathrm{polysaccharide}\mathrm{yield}\left(\%\right)=\frac{m}{M}\times 100\%$$

where, *m* is the mass of dried polysaccharide (g) and *M* is the mass of mulberry powder (g).

### 2.4. Characterization of MFP

#### 2.4.1. Chemical composition analysis

D-(+)-glucose was used as a standard and the total sugar content was determined by the phenol-sulfuric acid method (7). Bovine serum albumin (BSA) was used as a standard and the Bradford method was used to determine the total protein content (8). D-(+)-glucuronide was used as a standard and the total glucuronide content was determined by the carbazole-sulfuric acid method (9).

#### 2.4.2. Analysis of monosaccharide composition

The monosaccharide composition of MFP was detected by high-performance liquid chromatography using PMP (1-phenyl-3-methyl-5-pyrazolone) pre-column derivatization (6). Each MFP derivatized sample and reference solution were filtered through a 0.45 μm microporous membrane and then injected sequentially into an Agilent 1260 high-performance liquid chromatography (Agilent Technologies, USA). The sample was injected in a volume of 10 μL and detected by gradient elution on a ZORBOX SB-C18 (4.6 mm × 250 mm, 5 μm) column (The gradient elution conditions are shown in Supplementary Table 1) with a UV detector (wavelength 245 nm) using a mixture of phosphate buffer solution (A, 0.1 mol/L, pH 7.4) and acetonitrile (B) as the mobile phase.

#### 2.4.3. Fourier transform infrared spectroscopy (FI-IR) analysis

An appropriate amount of dried MFP sample was mixed with an appropriate amount of KBr in agate, pressed into pellets according to the KBr disc method, and analyzed using an IRAffinity-1S (Shimadzu Enterprise Management Co., LTD, China) infrared spectrometer in the range of 400–4000 cm^–1^ Infrared spectral scanning analysis was performed.

#### 2.4.4. Nuclear magnetic resonance spectroscopy (NMR) analysis

The 25 mg of dried MFP sample was fully dissolved in NMR tubes with 500 μL D_2_O, respectively, and then ^1^H NMR and ^13^C NMR were recorded with an AVANCE NEO 600 MHZ (Bruker, Germany) spectrometer with chemical shifts expressed in ppm.

#### 2.4.5. Thermogravimetric (TG) analysis

The thermal property of the MFP sample was studied using simultaneous thermal analysis (Q50 TGA, America). The heating rate was 10 °C/min, the heating range was 25–800°C, and the flow rate was 40 mL/min under a nitrogen atmosphere.

#### 2.4.6. Scanning electron microscopy (SEM) analysis

The surface morphology of each sample was observed by a scanning electron microscope (SEM, JSM-6490LV, Japan) at an accelerating voltage of 20 kV. Before observation, the dry sample was sputtered with a platinum layer, and SEM images were collected by a scanning electron microscope at 200 × magnification.

#### 2.4.7. Triple helix structure analysis

The present study refers to the method of Zhang et al. with slight modifications (10). A total of 1.5 mL of MFP solution (2.0 mg/mL) was mixed with 1.5 mL of Congo red solution (160 μg/mL), and 2 mol/L NaOH solution was added to bring the final NaOH concentration in the solution to 0, 0.1, 0.2, 0.3, 0.4, and 0.5 mol/L. The solution was left at room temperature for 10 min, and the maximum absorption wavelength (λ_max_) was measured with a SpectraMax Plus enzyme marker to determine the maximum absorption wavelength (λ_max_) in the range of 400–600 nm. The distillate water was used in place of the blank control.

### 2.5. Assay for antioxidant activity

#### 2.5.1. DPPH radical scavenging activity

The ability of MFP to scavenge DPPH radicals was based on published methods with some modifications (11). Briefly, 50 mg of MFP samples were dissolved in 100 mL of distilled water and diluted to obtain MFP polysaccharide solutions of different concentrations (0.1, 0.2, 0.3, 0.4, 0.5 mg/mL). Then, 1 mL of MFP solution with different concentrations was mixed with 1 mL of DPPH solution (0.2 mmol/L, dissolved in anhydrous ethanol), vortexed, and shaken, and the reaction was carried out for 30 min at room temperature and protected from light. Finally, the absorbance at 517 nm was measured with a microplate reader, and ascorbic acid was used as a positive control. The DPPH radical scavenging activity was calculated as follows:


$$\mathrm{DPPH}\mathrm{radical}\mathrm{scavenging}\mathrm{activity}\left(\%\right)=\left(1-\frac{A_{s}-A_{c}}{A_{b}}\right)\times 100\%$$

where *A*_*s*_ is the absorbance the of sample solution and DPPH solution, *A*_*c*_ is the absorbance of the mixed solution with anhydrous ethanol instead of DPPH solution, and *A*_*b*_ is the absorbance of the mixed solution in which distilled water replaces the sample solution.

#### 2.5.2. ABTS radical scavenging activity

The ability of MFP to scavenge ABTS radicals was determined according to the method reported in the literature, with slight modifications (12). The ABTS radical solution was prepared by mixing equal volumes of ABTS solution (7.00 mmol/L) and potassium persulfate solution (K_2_S_2_O_8_, 2.45 mmol/L) and reacting at room temperature for 12 h in the dark. The solution was then further diluted with distilled water so that the absorbance at 734 nm was 0.70 ± 0.02. A total of 3 mL of ABTS radical solution was mixed with 0.4 mL of MFP polysaccharide solution at different concentrations (0.1, 0.2, 0.3, 0.4, 0.5 mg/mL) and shaken. The reaction was carried out for 30 min at room temperature and protected from light, and the absorbance at 734 nm was measured with a microplate reader, using ascorbic acid as a positive control. The ABTS radical scavenging activity was calculated as follows:


$$\mathrm{ABTS}\mathrm{radical}\mathrm{scavenging}\mathrm{activity}\left(\%\right)=\left(1-\frac{A_{s}-A_{c}}{A_{b}}\right)\times 100\%$$

where *A*_*s*_ is the absorbance of the sample solution and ABTS radical solution, *A*_*c*_ is the absorbance of the mixed solution of ultrapure water instead of ABTS radical solution, and *A*_*b*_ is the absorbance of the mixed solution of distilled water instead of the sample solution.

### 2.6. Hypoglycemic activity assay

#### 2.6.1. α-glucosidase inhibition assay

The α-glucosidase inhibition activity assay of MFP was performed with some modifications based on the method previously reported method (13). Different concentrations (2, 4, 6, 8, and 10 mg/mL) of MFP solution, α-glucosidase (0.3 U/mL), and p-nitro-α-D-glucopyranoside solution (PNPG, 1.5 mmol/L) were prepared with phosphate buffer solution (0.1 mol/L, pH 6.9). A total of 50 μL of different concentrations of MFP solution and 100 μL of α-glucosidase were mixed and incubated for 20 min at 37°C. A total of 100 μL of PNPG was added and incubated for 10 min at 37°C. Finally, 1 mL of Na_2_CO_3_ (1 mol/L) was added to terminate the reaction, and its absorbance at 400 nm wavelength was measured with a microplate reader, and acarbose was used as a positive control. The α-glucosidase inhibition rate was calculated as follows:


$$\alpha -\text{glucosidas}e\text{inhibitio}n\text{rat}e\left(\%\right)=\left(1-\frac{A_{s}-A_{c}}{A_{b}}\right)\times 100\%$$

where *A*_*s*_ is the absorbance of sample solution, PNPG solution, and α-glucosidase solution; *A*_*c*_ is the absorbance of the mixture of buffer solution instead of the α-glucosidase solution, *A*_*b*_ is the absorbance of the mixture of buffer solution instead of sample solution.

#### 2.6.2. α-amylase inhibition assay

The *in vitro* α-amylase inhibition assay was modified according to the previously reported method (14). Briefly, MFP solution and α-amylase solution (1 U/mL) were first prepared in phosphate buffered solution (0.1 mol/L, pH 6.9) at different concentrations (2, 4, 6, 8, and 10 mg/mL), respectively. A total of 500 μL of different concentrations of MFP solution was thoroughly mixed with 500 μL of α-amylase solution and incubated at 37°C for 10 min. Then 500 μL of 1% (w/v) soluble starch solution was added, and the incubation was continued at 37°C for 10 min. At the end of the incubation, the reaction was terminated by adding 1 mL of DNS reagent and boiling it for 5 min. After cooling to room temperature, the solution was diluted to 10 mL with ultrapure water, and the absorbance at 520 nm was measured with a microplate reader and used acarbose as a positive control. The α-amylase inhibition rate was calculated as follows:


$$\alpha -\text{amylaseinhibitionrate}\left(\%\right)=\left(1-\frac{A_{s}-A_{c}}{A_{b}}\right)\times 100\%$$

where *A*_*s*_ is the absorbance of the mixed solution containing α-amylase solution, starch solution, and sample, *A*_*c*_ is the absorbance of the mixed solution with buffer solution instead of the α-amylase solution, *A*_*b*_ is the absorbance of the mixed solution with buffer solution instead of sample solution.

### 2.7. Statistical analysis

All indicators were analyzed using SPSS software for data analysis (20.0, IBM, USA) and expressed as mean ± standard deviation. Data were analyzed for significance using analysis of variance (ANOVA) and multiple comparisons between groups using the LSD method. *P* < 0.05 indicates a significant difference between groups and *P* < 0.01 indicates a highly significant difference between groups. All calculations were performed using IBM SPSS Statistics 26, Origin 2021 and GraphPad Prism 9 statistical software.

## 3. Results and discussion

### 3.1. Chemical composition of MFP

The Table 1 showed that the extraction method had a significant effect (*p* < 0.05) on the yield, total sugar content, protein content, and uronic acid content of the MFP. The yield of the six MFPs ranged from 2.10% to 13.32%. The high concentration of ethanol solvent combined with ultrasonic extraction may have contributed to the breakage and hydrolysis of the cell wall and promoted the diffusion of polysaccharides, resulting in MFP-III producing a larger yield than the other five methods. The total sugar content of MFP was reduced in the order MFP-III (92.21%) >MFP-IV (87.10%) >MFP-I (83.84%) >MFP-VI (81.28%) >MFP-V (66.07%) >MFP-II (64.33%) decreased in order, indicating that carbohydrates were the main component of the six MFPs. Due to the cavitation impact of ultrasound waves, which was able to disrupt the cell wall and cell membrane and encourage the leakage of intracellular chemicals, MFP-III and MFP-IV had much higher total sugar content than the other fractions (15). The lowest sugar content was found in MFP-II, which may have been caused by the prolonged high temperature and the breaking up of glycosidic linkages as well as the higher extraction temperature, which increased the diffusion of impurities and decreased the sugar content (16). Additionally, Table 1 demonstrates that all six MFPs had higher uronic acid content, with MFP-V (16.43%), MFP-IV (15.24%), MFP-I (14.09%), and MFP-VI (12.05%) having significantly higher uronic acid content than MFP-II (7.71%) and MFP -III (9.11%). This suggests that all six MFPs are acidic polysaccharides. Following TCA deproteinization, MFP-III and MFP-II had protein contents that were much lower than those of the other fractions at 0.60% and 0.97%, respectively, whereas MFP-V and MFP-IV had greater protein contents at 7.29% and 6.46%, respectively.

**TABLE 1:** The chemical compositions and monosaccharide composition of MFP obtained by different extraction methods.

Item	Sample					
	MFP-I	MFP-II	MFP-III	MFP-IV	MFP-V	MFP-VI
Yield (%)	11.06 ± 0.61^b^	6.08 ± 0.54^d^	13.32 ± 0.98^a^	4.00 ± 0.59^e^	2.10 ± 0.50^f^	8.45 ± 0.85^c^
Total sugar (%)	83.84 ± 0.24^c^	64.33 ± 0.43^f^	92.21 ± 0.66^a^	87.10 ± 0.27^b^	66.07 ± 0.06^e^	81.28 ± 0.32^d^
Protein (%)	2.74 ± 0.11^d^	0.97 ± 0.03^e^	0.60 ± 0.07^f^	6.46 ± 0.08^b^	7.29 ± 0.12^a^	3.30 ± 0.08^c^
Uronic acid (%)	14.09 ± 0.15^c^	7.71 ± 0.19^f^	9.11 ± 0.88^e^	15.24 ± 0.11^b^	16.43 ± 0.17^a^	12.05 ± 0.36^d^
**Monosaccharide composition (%)**						
Mannose	2.67	3.39	1.99	2.93	2.71	4.01
Rhamnose	9.17	5.58	2.98	6.33	12.65	7.78
Gluconic acid	nd	nd	0.41	4.44	1.42	nd
Galacturonic acid	0.8	1.16	nd	nd	11.49	0.53
Glucose	80.0	73.55	91.15	80.99	47.17	78.50
Xylose	3.35	3.61	3.09	3.43	12.51	4.68
Arabinose	4.09	12.71	0.39	1.86	12.05	4.49

The values are presented as mean ± SD (*n* = 3). Means within a row with different superscripts differ significantly (*P* < 0.05). nd, not detected.

### 3.2. Monosaccharide composition of MFP

**FIGURE 3:**
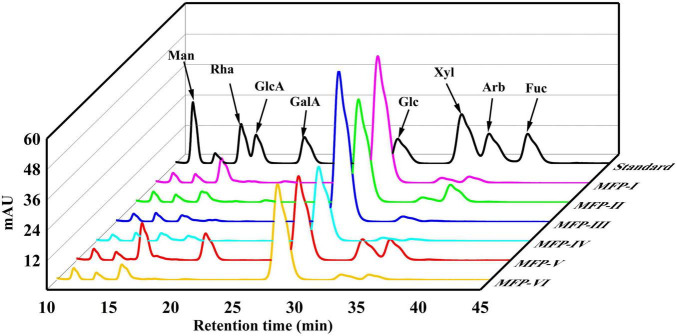
HPLC diagram of monosaccharide standard and different components of mulberry polysaccharides.

In this study, the PMP pre-column derivatization approach was used to ascertain the monosaccharide content of six MFPs. The monosaccharide composition of the MFP hydrolysate was identified by comparing the retention time with that of the standards. Table 1 and Figure 3 present the findings. Table 1 shows that the six MFPs were mostly composed of glucose (Glc), then mannose (Man), rhamnose (Rha), xylose (Xyl), and arabinose (Arb). The greatest Glc concentration was found in MFP-III (91.15%), MFP-II significantly increased the proportion of Arb (12.71%), and MFP-VI increased the content of Rha (7.78%). The hydrolytic breakage of more polysaccharide chains and the breaking of intermolecular hydrogen bonds as a result of large polar aqueous solvent-assisted sonication (17), which affects the monosaccharide composition, are likely to blame for the significantly higher content of Rha, Xyl, GlaA, and Arb in MFP-V than in the other fractions. The results of the current study are consistent with Chen et al. report’s that mulberry polysaccharides are made up of Gla, Arb, Glc, Xyl, and Man. Different extraction methods had little impact on the type of monosaccharides, but they did alter the ratio of monosaccharides with various physicochemical properties and potential variations in their biological activities (18).

### 3.3. FT-IR spectroscopy of MFP

**FIGURE 4:**
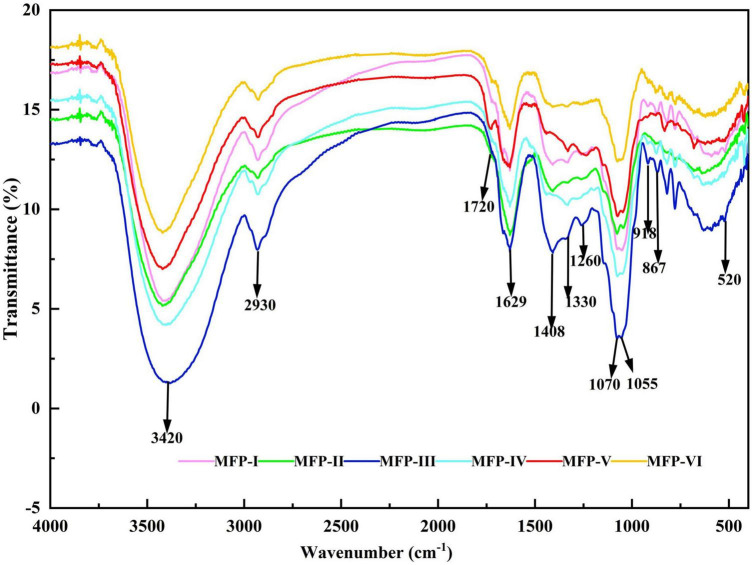
Infrared absorption spectra of mulberry polysaccharide samples prepared by different extraction methods.

In this study, the molecular structures of the six MFPs were analyzed using infrared spectroscopy. Six MFPs shown in Figure 4 exhibited the typical characteristic absorption peaks of polysaccharides in the range of 4000–400 cm^–1^. There is a broad strong absorption peak near 3420 cm^–1^, which is the O-H stretching vibration in the sugar residue, and the presence of a stronger absorption peak near 2930 cm^–1^ is a stretching vibration of the asymmetric C-H of the sugar, which may contain -CH_2_ or -CH_3_ (19). The absorption peak near 1720 cm^–1^ is considered to be the stretching vibration of the carboxyl group (-COOH) (20), which further proves that these MFPs are acidic polysaccharides and that the absorption peak of MFP-V is stronger here, indicating a higher content of glyoxylate acid, which is consistent with the results of 3.1. The absorption peaks presented at 1629 and 1408 cm^–1^ are symmetric and asymmetric stretching vibrations between C = O, while the absorption peak appearing near 1330 cm^–1^ is caused by the deformation vibrations of O-H (21). The absorption peak appearing between 1055 and 1070 cm^–1^ is the asymmetric stretching vibration between C-O-C, indicating that there are pyramidic bonds in six MFPs (22), and the absorption peak around 918 cm^–1^ further indicates that pyran presence of glucose groups. The absorption peaks of MFP-III, MFP-V, and MFP-I appearing near 1260 cm^–1^ are S = O stretching vibration (23), suggesting that these three components may contain sulfate radicals. In addition, the characteristic absorption peak located at 918 cm^–1^ was due to β-anomeric carbon (24), and the signal at 867 cm^–1^ is caused by the α-configuration glycosidic bond (25). The above results indicate that there is no significant effect of different extraction methods on the type of glycosidic bond and conformation of MFP. The obtained polysaccharides all contained α-configuration glycosidic bonds and β-configuration glycosidic bonds, and they are all in the form of pyranose, which was consistent with the results of Liu et al. (26).

### 3.4. NMR spectroscopy of MFP

**FIGURE 5:**
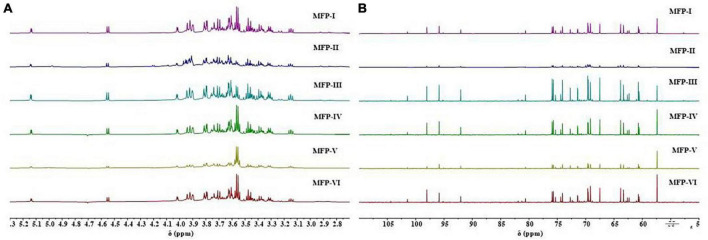
^1^H-NMR plots **(A)** and ^13^C-NMR plots **(B)** of mulberry polysaccharide samples prepared by different extraction methods.

The chemical shifts of protons on the glycosyl ring carbon (C-2 to C-6) of polysaccharides are usually in the range of 3.3–4.5 ppm due to the shielding effect of hydroxyl groups. The proton signals in this interval overlap severely and are not favorable for analysis, but the chemical shift of hydrogen protons (H-1) on the polysaccharide anomeric carbon (C-1) is in the lower field, usually in the range of 4.5–5.5 ppm, which facilitates structural analysis (11). In general, the chemical shift of H-1 of α-pyranose is greater than 4.95 ppm, the chemical shift of H-1 of β-pyranose is less than 4.95 ppm, and the chemical shift of H-1 of furanose is about 5.4 ppm, by which the conformation of the sugar ring can be determined (25). The ^1^H-NMR spectra of the six MFPs are shown in Figure 5A, in which anomeric proton signals are observed near 4.55 ppm and 5.15 ppm, indicating that the six MFPs contain both α-glucopyranose and β-glucopyranose, and there is no chemical shift near 5.4 ppm, indicating the absence of furanose, which is consistent with the results of the IR spectrum of 3.3. A total of 3.3–4.0 ppm range is the chemical shift of protons (H-2∼H-6) on the C-2∼C-6 of the MFP sugar ring, the strong absorption peak near 3.5 ppm is the proton peak of methylene, and 4.7 ppm is the chemical shift of solvent D_2_O. However, the ratios of the two configurations obtained by different methods are different. Judging from the intensity of the heterotopic proton outgoing peaks at the same chemical shifts, MFP-I, MFP-III, MFP-IV, and MFP-VI exist mainly in the β-configuration, MFP-II mainly in the α-configuration, while the ratio of β-configuration to α-configuration in MFP-V does not differ much.

In ^13^C-NMR, the carbon signals of polysaccharides are usually distributed between 60 and 110 ppm, the positions of C-2 to C-6 resonance absorption peaks on the sugar ring are roughly 60–90 ppm, and the positions of the resonance absorption peaks of the anomeric carbon (C-1) are roughly 90–110 ppm. If the signal shift lies at 103–110 ppm, it is a β-type glycosidic bond and 90–103 ppm is an α-type glycosidic bond (11). As shown in Figure 5B, in the ^13^C-NMR spectra of the six MFPs, the anomeric carbon signal region is concentrated in the range of 90–105 ppm, indicating the presence of both α-type and β-type glycosidic bonds in the six MFPs, which is consistent with the ^1^H-NMR results. In the range of 90–105 ppm, the six MFPs had mainly four absorption peaks (101.46, 98.04, 95.30, 92.05 ppm) indicating the presence of mainly four sugar residues. The peak abundances of MFP-I, MFP-III, MFP-IV, and MFP-VI are significantly higher than those of MFP-II and MFP-V, indicating that the ratios of glycosidic bonds obtained by different extraction methods are different, which are consistent with the ^1^H-NMR results. In addition, the peaks at 101.0 and 104.0 ppm are attributed to β-Man and β-Glc, respectively (27), the apparent absorption peak at δ 55.74 ppm is that of carbon on methylene, and the signal near 60 ppm is generated by the unsubstituted C-6 resonance of glucosyl and mannosyl residues (28). The anomeric carbon at around 98.04 ppm resonance is attributed to the C-1 group of glucopyranose (Glcp), indicating the presence of α-D-Glcp, while the peak at 104.40 ppm suggests the possible presence of β-D-Galp (29). The signal at 82–84 ppm was reported to correspond to the chemical shifts of C-4 and C-5 of furanose (30), while the six MFPs had no chemical shifts at 82–88 ppm, indicating the absence of furanose, in agreement with the results of the ^1^H-NMR analysis.

### 3.5. Thermogravimetric (TG) analysis of MFP

**FIGURE 6:**
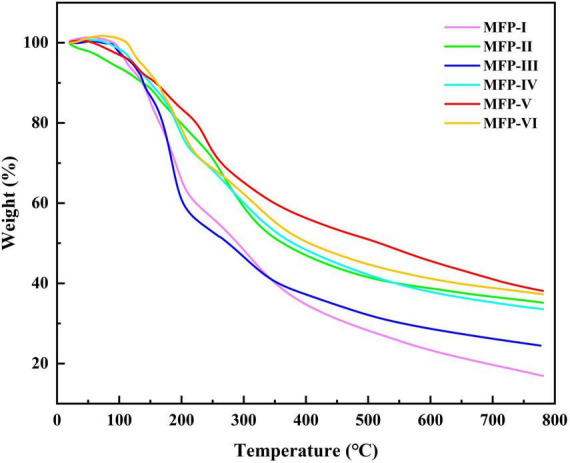
TG curves of mulberry polysaccharide samples prepared by different extraction methods.

The thermal stability of polysaccharides can affect their application in the food and pharmaceutical industries. Thermogravimetric analysis (TGA) is a commonly used method to determine thermal stability and degradation temperature, which is related to the variation of polysaccharide dehydration and decomposition processes with time and temperature (31). The thermograms of MFP obtained by different extraction methods are shown in Figure 6. With the increase in temperature, the weight loss of the six MFPs were enhanced and the TG curves were similar in shape, mainly showing a three-step degradation pattern. The first stage of mass loss occurred in the temperature range of 30–120°C, which may be related to the evaporation of free and bound water in the polysaccharide samples (32). The second stage of weight loss changes in the range of 200–500°C, which is mainly caused by the loss of crystalline water in the polysaccharide samples and the depolymerization and cleavage of polysaccharide structures, including the breakage of C-C and C-O bonds (24). In this temperature range, the mass loss rate was the highest for MFP-I (83.371%) and the lowest for MFP-V (62.043%). There were subtle differences in thermal stability and degradation behavior of the different MFP samples, which may be due to the different internal structures of the polysaccharides obtained from the six different extraction methods. When the temperature was further increased to 600 °C, the mass loss rates of the six MFPs polysaccharides gradually decreased, and the final polysaccharide residual mass percentages were in the following order from high to low: MFP-V (37.957%) >MFP-VI (37.094%) >MFP-II (35.083%) >MFP-IV (33.446%) >MFP-III (24.461%) >MFP-I (16.629%). These findings suggested that although there were some variations in the quantity and rate of mass loss, the thermodynamic curves of polysaccharides from various extraction methods exhibited comparable tendencies. This difference in thermal stability may be related to the extraction method, chemical composition, and structure of polysaccharides.

### 3.6. Scanning electron microscopy (SEM) analysis of MFP

**FIGURE 7:**
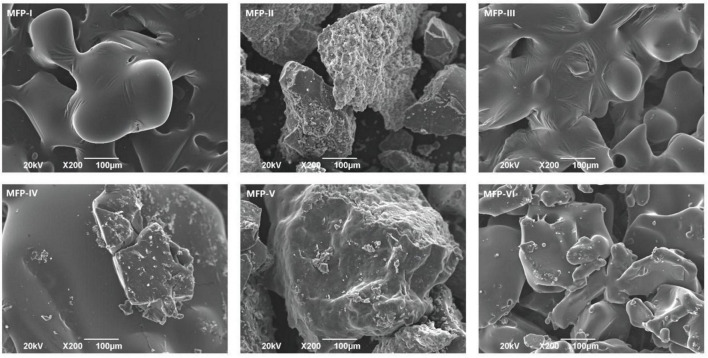
SEM images of mulberry polysaccharide samples prepared by different extraction methods.

Scanning electron microscopy (SEM) is an important method to detect the morphological characteristics of polysaccharide micromorphology. As shown in Figure 7, the MFPs extracted by different methods have different sizes and shape structures. The surfaces of both MFP-I and MFP-III were thin film-like and contained a few folds. At 200 × magnification, the surface of MFP-III had a dense morphology, while MFP-I is looser. This may be due to the high polysaccharide content in MFP-III, which had a high degree of molecular cross-linking within the polysaccharide and a tight structure. MFP-II and MFP-V had a lower polysaccharide content and consisted of irregular and rough surfaces with small particles of different shapes. MFP-II consisted of more particles and had a loose structure, which may be due to the damage caused to the cellular tissues of the raw mulberry by the long-time high-temperature extraction, thus destroying the polysaccharide structure. MFP-IV and MFP-VI had sheet-like dense morphology, relatively smooth surface structure, and stable sheet-like structure. At low multiplicity, MFP-VI molecules were tightly arranged and had higher polysaccharide content. This may be because hot water extraction promotes the degradation and rupture of the cell wall, which in turn enhances the release of polysaccharides. The differences in the microstructure of the six MFPs suggested that different extraction methods have different effects on the surface morphology of polysaccharides, which may be related to the fact that polysaccharides have different physical properties, such as polysaccharide content and viscosity. These different structures could further affect the biological activity of polysaccharides and their applications in the food and pharmaceutical industries.

### 3.7. Triple helix structure analysis of MFP

**FIGURE 8:**
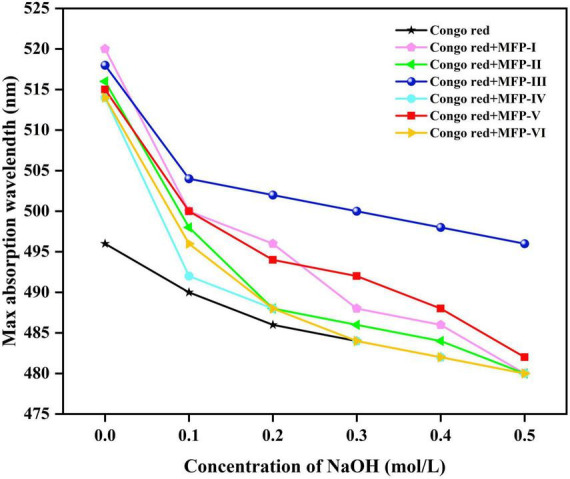
Analysis of spiral-curl transformation of mulberry polysaccharides in different NaOH concentrations.

It was found that polysaccharides with a triple helix structure would complex with Congo red and increase their maximum absorption wavelength; however, as the pH of the solution increases, the triple helix conformation of the polysaccharide will gradually change to a single coiled conformation, reducing the occurrence of complexation reaction and thus decreasing the maximum absorption wavelength (33). Therefore, the Congo red experiments are commonly used to study the conformation of polysaccharides. As shown in Figure 8, the maximum absorption wavelength of Congo red solution without polysaccharides was 496 nm. In this study, the maximum absorption wavelength of congo red solution of six MFPs. MFP-I, MFP-II, MFP-III, MFP-IV, MFP-V, and MFP-VI were higher than those in congo red solution, and the maximum absorption wavelength gradually increased in the process of NaOH concentration decreased, indicating that MFP-I, MFP-II, MFP-III, MFP-IV, MFP-V, and MFP-VI do not have triple helix structure.

### 3.8. Assay for antioxidant activity

#### 3.8.1. DPPH radical scavenging activity

**FIGURE 9:**
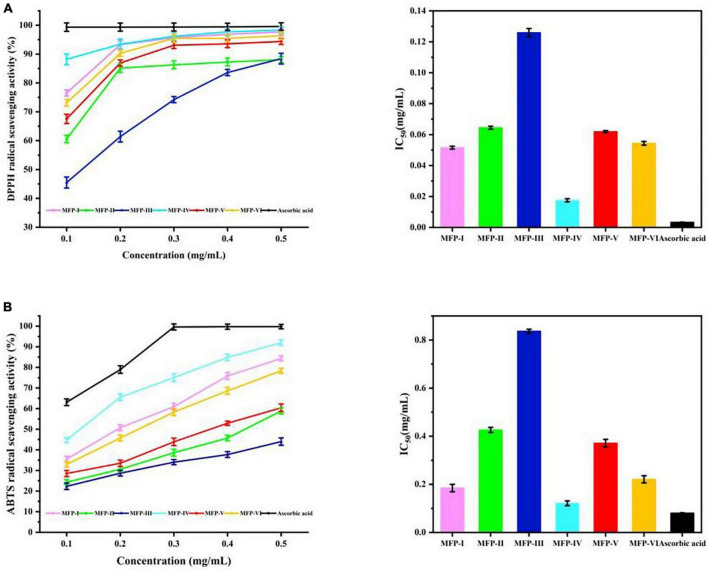
*In vitro* antioxidant activity of MFP: **(A)** DPPH radical scavenging rate; **(B)** ABTS radical scavenging.

Excessive free radical production can lead to oxidative damage in the body and induce cardiovascular diseases, central nervous system disorders, renal failure, and other diseases (34). Polysaccharides contain functional groups with reducing properties such as hydroxyl and aldehyde groups, which allow them to be developed as natural antioxidants to inhibit cellular aging and improve symptoms of degenerative diseases. DPPH, as a stable nitrogen-centered lipophilic free radical, is an effective agent to evaluate the radical scavenging activity of antioxidant materials (35). As shown in Figure 9A, within a certain concentration range (0.1–0.5 mg/mL), all six mulberry polysaccharide fractions showed a concentration-dependent increase in the scavenging rate of DPPH radicals, and the scavenging ability was MFP-IV > MFP-I > MFP-VI > MFP-V > MFP-II > MFP-III. At 0.5 mg/mL concentration, the clearance rate of MFP-IV reached 98.32%, which was similar to that of ascorbic acid at the same concentration, but the clearance rate of MFP-III was only 88.42%. The IC_50_ values of MFP-I, MFP-II, MFP-III, MFP-IV, MFP-V, MFP-VI, and ascorbic acid (Supplementary Table 2) were calculated to be 0.052, 0.065, 0.126, 0.018, 0.062, 0.054, and 0.003 mg/mL, respectively, and the samples with lower IC_50_ value have higher DPPH scavenging ability. Therefore, MFP-IV had a stronger ability to scavenge DPPH radicals within a certain concentration range. It has been shown that uronic acid and the quantity of unmethylated carboxyl groups, which produce hydrogen ions (H^+^) and react with DPPH free radical solution to generate more stable free radicals, are associated to the antioxidant mechanism of polysaccharides (36). According to this study, the amount of uronic acid in MFP-IV and MFP-III may be associated to the difference in DPPH radical scavenging activity between them; the more uronic acid, the higher the polysaccharide radical scavenging rate.

#### 3.8.2. ABTS radical scavenging activity

The ability to scavenge ABTS radicals can indirectly reflect the antioxidant effect of the substance on non-physiological free radicals generated by pesticide pollution, smoke, tobacco smoke, and chemical agents (16). As shown in Figure 9B, within a certain concentration range (0.1–0.5 mg/mL), all six MFPs showed significant ABTS radical scavenging effects, the scavenging ability was concentration-dependent, and the scavenging activities showed highly significant differences between the fractions (*P* < 0.01), with the scavenging ability in the order of MFP-IV > MFP-I > MFP-VI > MFP-V > MFP-II > MFP-III. At the concentration of 0.5 mg/mL, the clearance rate of MFP-IV could reach 92.01%, MFP-I, MFP-VI, and MFP-V could reach 84.35%, 78.36%, and 60.4%, respectively, while the clearance of MFP-III was the lowest at 44.01%. By calculating the IC_50_ values (Supplementary Table 3), it can be seen that the IC_50_ values of MFP-IV and MFP-III were 0.122 and 0.837 mg/mL, respectively, which showed that the scavenging ability of MFP-IV for ABTS radicals was significantly better than the other fractions, which is consistent with the results of DPPH study. As shown in Supplementary Table 4 for different MFP samples, the ABTS radical scavenging activity (y) showed a quadratic relationship with concentration (x) (*P* < 0.05). Polysaccharides can degrade due to their high water solubility and the ultrasound-assisted extraction process. The chemical groups in the degraded samples produced better water solubility and a larger surface area, making them more prone to contact with free radicals and possessing stronger antioxidant activity (37). MFP-IV has superior antioxidant activity because polysaccharides with a high uronic acid concentration have higher biological activity, which is also greatly influenced by the monosaccharide composition of the polysaccharides.

### 3.9. Hypoglycemic activity assay

#### 3.9.1. α-glucosidase inhibition assay

**FIGURE 10:**
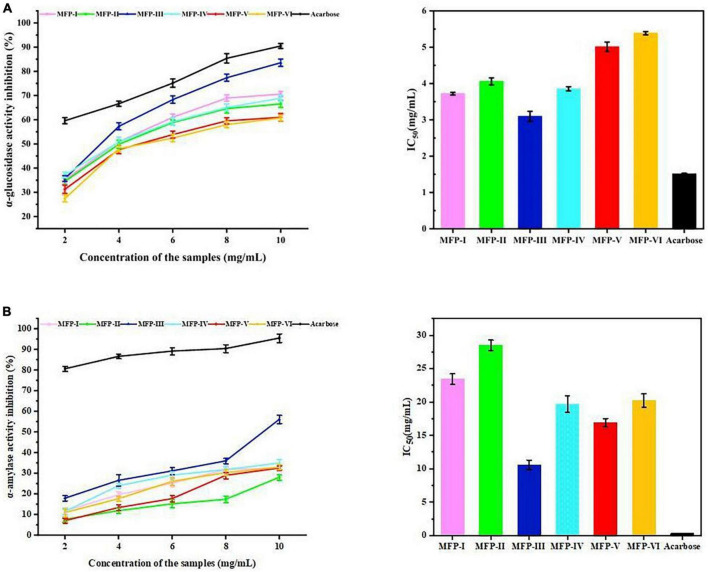
Hypoglycemic effects of MFP *in vitro*: **(A)** inhibitory effect on α-glucosidase; **(B)** inhibitory effect on α-amylase activity.

According to several reports, α-amylase and α-glucosidase are the primary enzymes influencing post-prandial hyperglycemia. As a result, inhibiting the activities of α-amylase and α-glucosidase can significantly lower post-prandial blood glucose levels, which is beneficial for the management and prevention of type II diabetes (38). Figure 10A displays the inhibitory effects of six MFPs on α-glucosidase activity. The outcomes demonstrated that, within a specific concentration range of 2 to 10 mg/mL, the effects of the six MFPs on α-glucosidase inhibition were concentration-dependent. MFP-III and MFP-I showed inhibition rates on α-glucosidase activity of 70.61% and 83.56%, respectively, whereas MFP-V and MFP-VI, had the lowest activity, 61.01% and 60.74%, respectively. The six MFPs inhibited α-glucosidase activity in the following order: MFP-III > MFP-I > MFP-IV > MFP-II > MFP-V > MFP-VI. In addition, MFP-III showed the strongest inhibition of α-glucosidase with an IC_50_ value (Supplementary Table 5) of 3.094 mg/mL (*P* < 0.05), and MFP-VI showed the weakest inhibition with an IC_50_ value of 5.385 mg/mL (*P* < 0.05), this phenomenon may be related to the content of glucose and uronic acid in MFP. The polysaccharide concentration (x) of different MFP showed a quadratic relationship (*P* < 0.05) with the α-glucosidase activity (y), and the results are shown in Supplementary Table 6. Previous research has demonstrated that the hydrogen bonds formed by the -OH and -COOH groups on polysaccharide branched chains and α-glucosidase residues result in the suppression of enzyme activity (24). As a result of the exposure of more active sites, the greater polysaccharide content of MFP-III and MFP-IV and the higher uronic acid content of MFP-IV and MFP-I in this study are thought to have stronger glucose-lowering activity.

#### 3.9.2. α-amylase inhibition assay

Figure 10B illustrates the inhibition of α-amylase activity by different mulberry polysaccharide fractions. The results showed that in a certain concentration range (2–10 mg/mL), the inhibition of α-amylase activity by the six MFPs increased with the increase of polysaccharide concentration. The highest rate of inhibition of α-amylase by MFP-III was 50.73%, whereas MFP-II and MFP-V had the lowest rates at 27.51% and 26.43%, respectively. The magnitude of the inhibitory ability of the six MFPs on α-amylase activity was in the following order: MFP-III > MFP-V > MFP-IV > MFP-VI > MFP-I > MFP-II with IC_50_ values (Supplementary Table 7) of 10.583, 16.903, 19.653, 20.227, 23.367, and 28.523 mg/mL, respectively (*P* < 0.05). The polysaccharide concentration (x) of MFP showed a quadratic relationship (*P* < 0.05) with α-amylase activity (y), and the results are shown in Supplementary Table 8. At a concentration of 10 mg/mL, MFP-III showed significant inhibition of α-amylase relative to the other fractions, which is consistent with the results of α-glucosidase studies. In addition, both MFP-V and MFP-IV have strong inhibitory effects on α-amylase, which may be related to the complex composition of monosaccharides and the content of uronic acid.

Although all of the six MFPs in this investigation exhibited lesser inhibition than acarbose, they still hold a lot of promise for the prevention and treatment of diabetes since they significantly inhibited α-amylase and α-glucosidase with an increase in concentration. In the treatment of type II diabetes, acarbose has a potent inhibitory action on starch hydrolase, which causes an accumulation of digested carbs that can be fermented by bacteria in the colon and result in side effects including bloating (31). Therefore, the search for a new inhibitor will help in the development of hypoglycemic drugs.

## 4. Conclusion

This study evaluated the effects of six extraction methods on the chemical composition, monosaccharide composition, structural characteristics, antioxidant activity, and hypoglycemic activity of MFP. The findings demonstrated that all six MFPs shared nuclear magnetic spectra and typical polysaccharide infrared spectral characteristics, and that the various extraction methods had little impact on their thermodynamic characteristics and similar trends. Additionally, none of the six MFPs contained triple helix structures. However, their chemical composition, monosaccharide concentration, and microscopic morphology were significantly influenced by the extraction methods. As a result of these structural variations, the MFP engaged in several biological activities. Due to its high uronic acid concentration, MFP-IV showed excellent antioxidant activity, while MFP-III had the most notable hypoglycemic action due to its largest polysaccharide content. The results of this study showed that there were significant differences in the chemical structure and biological activities of mulberry polysaccharides between different extraction methods, and the differences in their antioxidant and hypoglycemic activities were the result of the combined effect of various factors, which can provide important references for the application of mulberry polysaccharides in food and pharmaceutical industries.

## Data availability statement

The original contributions presented in this study are included in the article/Supplementary material, further inquiries can be directed to the corresponding authors.

## Author contributions

YH: investigation, methodology, and writing–original draft. WX: software and data curation. TT: investigation. HC: supervision and project administration. XZ: supervision, project administration, and funding acquisition. All authors have read and agreed to the published version of the manuscript.
