# Supplementary material for: Structural characteristics, antioxidant and hypoglycemic activities of polysaccharides from Mori Fructus based on different extraction methods
Source: Front Nutr. 2023 Apr 6;10:1125831. doi: 10.3389/fnut.2023.1125831 (PMC10117789; doi:10.3389/fnut.2023.1125831)
Supplement: Supplementary file 1 [file Data_Sheet_1.docx]

***Supplementary material***

**Structural characteristics, antioxidant, and hypoglycemic activities of polysaccharides from *Mori Fructus* based on different extraction methods**

**Yuanyuan Huang, Wen Xie, Ting Tang, Huaguo Chen*, Xin Zhou***

*** Corresponding authors:**

Xin Zhou, E-mail: alice9800@sina.com.

Huaguo Chen, E-mail: [chenhuaguo1981@163.com](mailto:chenhuaguo1981@163.com).

**Contents**

- [Figure S1](#图1). Standard curve.
- [Figure S2](#图2). ^1^H-NMR spectrum of six MFP.
- [Figure S3](#图3). ^13^C-NMR spectrum of six MFP.
- [Figure S4.](#图4) TGA thermograms of six MFP.
- [Table S1](#表1). Gradient elution conditions of mobile phase.
- [Table S2](#表2). Comparative analysis of different extraction methods on IC_50_ by LSD (DPPH).
- [Table S3](#表3). Comparative analysis of different extraction methods on IC_50_ by LSD (ABTS).
- [Table S4](#表4). Second-order polynomial of MFP polysaccharide concentration and ABTS radical scavenging activity.
- [Table S5](#表5). Comparative analysis of different extraction methods on IC_50_ by LSD (α -glucosidase).
- [Table S6](#表6). Second-order polynomial of MFP polysaccharide concentration and α -glucosidase inhibitory activity.
- [Table S7](#表7). Comparative analysis of different extraction methods on IC_50_ by LSD (α-amylase).
- [Table S8](#表8). Second-order polynomial of MFP polysaccharide concentration and α-amylase inhibitory activity.

**A**

**B**

**C**

**Figure S1** Standard curve (A: Standard curve of Glucose, B: Standard curve of protein, C: Standard curve of uronic acid).

**A B**

**C D**

**E F**

**Figure S2** ^1^H-NMR spectrum of six MFP (A, B, C, D, E, F).

**A B**

**C D**

**E F**

**Figure S3** ^13^C-NMR spectrum of six MFP (A, B, C, D, E, F).

**A B**


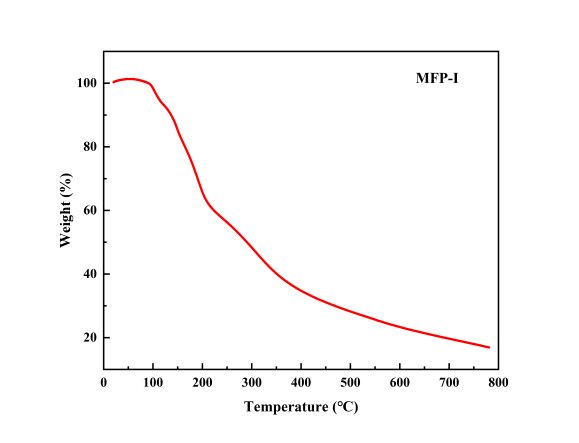

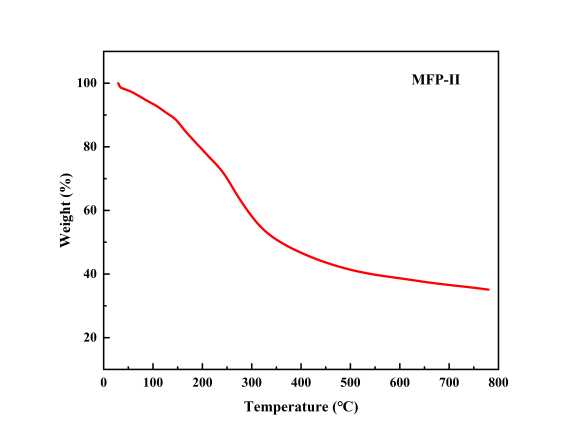


**C D**


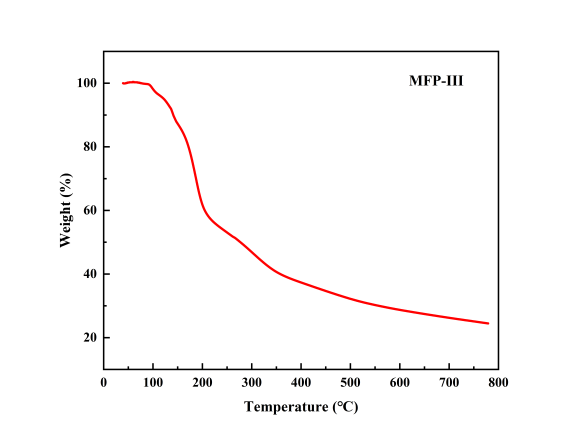

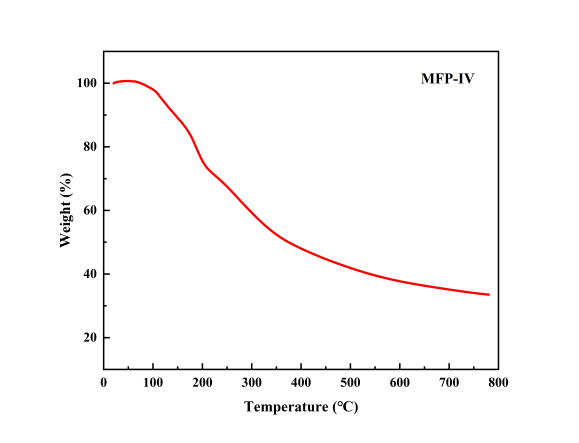


**E F**


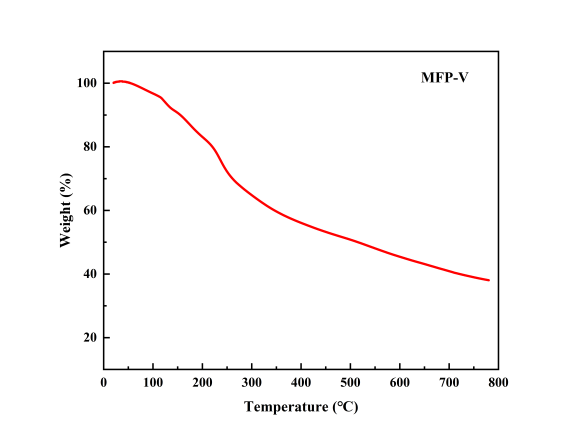

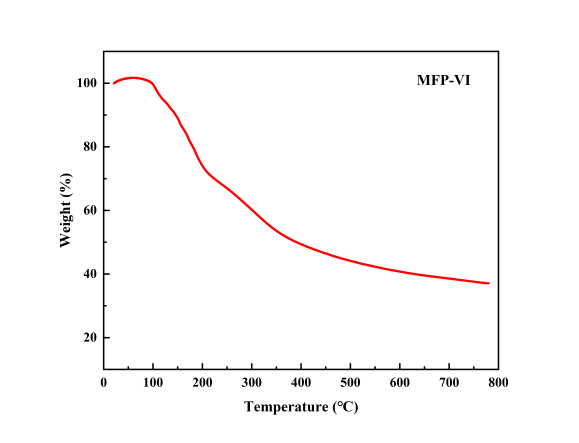


**Figure S4** TG thermograms of six MFP (A, B, C, D, E, F).

**Table S1** Gradient elution conditions of mobile phase.

| **Time/min** | **0** | **28** | **45** | **48** | **55** |
| --- | --- | --- | --- | --- | --- |
| A% | 84 | 84 | 81 | 75 | 75 |
| B% | 16 | 16 | 19 | 25 | 25 |

**Table S2** Comparative analysis of different extraction methods on IC_50_ by LSD (DPPH).

| **Samples** | **IC_50_** | ***X*_i_*-X*_VC_** | ***X*_i_*-X*_MFP-IV_** | ***X*_i_*-X*_MFP-I_** | ***X*_i_*-X*_MFP-VI_** | ***X*_i_*-X*_MFP-V_** | ***X*_i_*-X*_MFP-II_** |
| --- | --- | --- | --- | --- | --- | --- | --- |
| MFP-III | 0.1259 | 0.1225^ab^ | 0.1084^ab^ | 0.0744^ab^ | 0.0715^ab^ | 0.0640^ab^ | 0.0614^ab^ |
| MFP-II | 0.0645 | 0.0611^ab^ | 0.0470^ab^ | 0.0130^ab^ | 0.0101^ab^ | 0.0026^a^ |  |
| MFP-V | 0.0619 | 0.0585^ab^ | 0.0444^ab^ | 0.0104^ab^ | 0.0075^ab^ |  |  |
| MFP-VI | 0.0544 | 0.0510^ab^ | 0.0369^ab^ | 0.0029^a^ |  |  |  |
| MFP-I | 0.0515 | 0.0481^ab^ | 0.0340^ab^ |  |  |  |  |
| MFP-IV | 0.0175 | 0.0141^ab^ |  |  |  |  |  |
| Ascorbic acid | 0.0034 |  |  |  |  |  |  |

Data were presented as mean ± SD (n=3). ^a, b^ designate P < 0.05 and P < 0.01 between them, respectively.

**Table S3** Comparative analysis of different extraction methods on IC_50_ by LSD (ABTS).

| **Samples** | **IC_50_** | ***X*_i_*-X*_VC_** | ***X*_i_*-X*_MFP-IV_** | ***X*_i_*-X*_MFP-I_** | ***X*_i_*-X*_MFP-VI_** | ***X*_i_*-X*_MFP-V_** | ***X*_i_*-X*_MFP-II_** |
| --- | --- | --- | --- | --- | --- | --- | --- |
| MFP-III | 0.8368 | 0.7562^ab^ | 0.7152^ab^ | 0.6520^ab^ | 0.6157^ab^ | 0.4656^ab^ | 0.4105^ab^ |
| MFP-II | 0.4263 | 0.7562^ab^ | 0.3047^ab^ | 0.2415^ab^ | 0.2052^ab^ | 0.0551^ab^ |  |
| MFP-V | 0.3712 | 0.2907^ab^ | 0.2496^ab^ | 0.1864^ab^ | 0.1501^ab^ |  |  |
| MFP-VI | 0.2211 | 0.1405^ab^ | 0.0995^ab^ | 0.0363^ab^ |  |  |  |
| MFP-I | 0.1848 | 0.1043^ab^ | 0.0632^ab^ |  |  |  |  |
| MFP-IV | 0.1216 | 0.0410^ab^ |  |  |  |  |  |
| Ascorbic acid | 0.0805 |  |  |  |  |  |  |

Data were presented as mean ± SD (n=3). **^a, b^** designate P < 0.05 and P < 0.01 between them, respectively.

**Table S4** Second-order polynomial of MFP polysaccharide concentration and ABTS radical scavenging activity.

| **Samples** | **Second-order polynomial** | **R^2^** |
| --- | --- | --- |
| MFP-I | y = −61.3574x^2^+159.50429x+20.382 | 0.9916 |
| MFP-II | y = 91.64286x^2^+29.24429x+20.764 | 0.9914 |
| MFP-III | y = −12.78571x^2^+60.10143x+16.722 | 0.9880 |
| MFP-IV | y = −191.71429x^2^+228.76857x+24.886 | 0.9845 |
| MFP-V | y = 25.85714x^2^+67.68571x+20.694 | 0.9844 |
| MFP-VI | y = −60.28571x^2^+149.91143x+18.458 | 0.9997 |

**Table S5** Comparative analysis of different extraction methods on IC_50_ by LSD (α-glucosidase).

| **Samples** | **IC_50_** | ***X*_i_*-X*_Acarbose_** | ***X*_i-_*X*_MFP-III_** | ***X*_i_*-X*_MFP-I_** | ***X*_i_*-X*_MFP-IV_** | ***X*_i_*-X*_MFP-II_** | ***X*_i_*-X*_MFP-V_** |
| --- | --- | --- | --- | --- | --- | --- | --- |
| MFP-VI | 5.3853 | 3.8757^ab^ | 2.2910^ab^ | 1.6637^ab^ | 1.5313^ab^ | 1.3270^ab^ | 0.3740^ab^ |
| MFP-V | 5.0113 | 3.5017^ab^ | 1.9170^ab^ | 1.2897^ab^ | 1.1573^ab^ | 0.9530^ab^ |  |
| MFP-II | 4.0583 | 2.5487^ab^ | 0.9640^ab^ | 0.3367^ab^ | 0.2043^a^ |  |  |
| MFP-IV | 3.8540 | 2.3443^ab^ | 0.7597^ab^ | 0.1323 |  |  |  |
| MFP-I | 3.7217 | 2.2120^ab^ | 0.6273^ab^ |  |  |  |  |
| MFP-III | 3.0943 | 1.5847^ab^ |  |  |  |  |  |
| Acarbose | 1.5097 |  |  |  |  |  |  |

Data were presented as mean ± SD (n=3). **^a, b^** designate P < 0.05 and P < 0.01 between them, respectively.

**Table S6** Second-order polynomial of MFP polysaccharide concentration and α-glucosidase inhibitory activity.

| **Samples** | **Second-order polynomial** | **R^2^** |
| --- | --- | --- |
| MFP-I | y = −0.54071x^2^+10.93157x+15.54 | 0.9981 |
| MFP-II | y = −0.53857x^2^+10.40186x+16.158 | 0.9975 |
| MFP-III | y = −0.58607x^2^+12.82386x+13.29 | 0.9899 |
| MFP-IV | y = −0.39661x^2^+8.64179x+21.854 | 0.9964 |
| MFP-V | y = −0.54196x^2^+10.07607x+14.046 | 0.9793 |
| MFP-VI | y = −0.61571x^2^+11.21857x+9.094 | 0.9303 |

**Table S7** Comparative analysis of different extraction methods on IC50 by LSD (α-amylase).

| **Samples** | **IC_50_** | ***X*_i_*-X*_Acarbose_** | ***X*_i-_*X*_MFP-III_** | ***X*_i_*-X*_MFP-V_** | ***X*_i_*-X*_MFP-IV_** | ***X*_i_*-X*_MFP-VI_** | ***X*_i_*-X*_MFP-I_** |
| --- | --- | --- | --- | --- | --- | --- | --- |
| MFP-II | 28.5233 | 28.2008^ab^ | 17.9400^ab^ | 11.6200^ab^ | 8.8700^ab^ | 8.2966^ab^ | 5.0866^ab^ |
| MFP-I | 23.4367 | 23.1142^ab^ | 12.8534^ab^ | 6.5334^ab^ | 3.7834^ab^ | 3.2100^ab^ |  |
| MFP-VI | 20.2267 | 19.9042^ab^ | 9.6434^ab^ | 3.3234^ab^ | 0.5734^a^ |  |  |
| MFP-IV | 19.6533 | 19.3308^ab^ | 9.0700^ab^ | 2.7500^ab^ |  |  |  |
| MFP-V | 16.9033 | 16.5808^ab^ | 6.3200^ab^ |  |  |  |  |
| MFP-III | 10.5833 | 10.2608^ab^ |  |  |  |  |  |
| Acarbose | 0.3225 |  |  |  |  |  |  |

Data were presented as mean ± SD (n=3). **^a, b^** designate P < 0.05 and P < 0.01 between them, respectively.

**Table S8** Second-order polynomial of MFP polysaccharide concentration and α-amylase inhibitory activity.

| **Samples** | **Second-order polynomial** | **R^2^** |
| --- | --- | --- |
| MFP-I | y = −0.23518x^2^+5.51564x+1.428 | 0.9825 |
| MFP-II | y = 0.21357x^2^−0.25386x+8.156 | 0.9149 |
| MFP-III | y = 0.41518x^2^−0.69064x+19.418 | 0.9037 |
| MFP-IV | y = −0.37643x^2^+7.25014x−0.69 | 0.9638 |
| MFP-V | y = 0.02536x^2^+3.02971x+0.638 | 0.9515 |
| MFP-VI | y = −0.22018x^2^+5.47764x+0.41 | 0.9877 |
